# miR-3178 inhibits cell proliferation and metastasis by targeting Notch1 in triple-negative breast cancer

**DOI:** 10.1038/s41419-018-1091-y

**Published:** 2018-10-17

**Authors:** Peng Kong, Lie Chen, Muxin Yu, Jing Tao, Jiawei Liu, Yue Wang, Hong Pan, Wenbin Zhou, Shui Wang

**Affiliations:** Department of Breast Surgery, The First Affiliated Hospital with Nanjing Medical University, 300 Guangzhou Road, 210029 Nanjing, Jiangsu China

## Abstract

Triple-negative breast cancer (TNBC) has a poorer outcome than other subtypes of breast cancer, and the discovery of dysregulated microRNA (miRNA) and their role in tumor progression has provided a new avenue for elucidating the mechanism involved in TNBC. In this study, we identified that miR-3178 was significantly reduced in TNBC, and the low miR-3178 expression correlated with poor overall survival in TNBC but not in non-TNBC. The ectopic overexpression of miR-3178 suppressed TNBC cell proliferation, invasion, and migration by inhibiting the epithelial-to-mesenchymal (EMT) transition. Notch1 was validated as the direct target gene of miR-3178, which was confirmed by the dual-luciferase reporter assay. miR-3178 decreased the expression of Notch1 and restoration of Notch1 expression attenuated the inhibitory effects of miR-3178 on cell proliferation, metastasis, and the EMT in TNBC. miR-3178 inhibited cell proliferation and metastasis by targeting Notch1 in TNBC, and the restoration of miR-3178 might be a potential therapeutic strategy for TNBC.

## Introduction

Triple-negative breast cancer (TNBC) represents an aggressive subtype of breast cancer characterized by lack of the estrogen receptor, progesterone receptor, and human epidermal growth factor receptor 2 (HER2)^1^. This subtype of patient does not benefit from endocrine and anti-HER2 therapy. Chemotherapy is the common option for adjuvant therapy. TNBC patients have a poorer prognosis than those with other subtypes of breast cancer^2,3^. This may be partly due to the inherently aggressive clinical behaviors and lack of proper therapeutic targets^2,4^, as well as the high heterogeneity in genomics, epigenomics, transcriptomics, and proteomics that characterize TNBC molecular subtypes^5–7^. For better treatment of TNBC, there is an urgent need to further understand the biological characteristics of this disease and to identify novel therapeutic targets.

The underlying cause of death in majority of breast cancer patients is metastasis, of which the main characteristic is the epithelial-to-mesenchymal transition (EMT)^8^. The EMT acts as the initial event during tumor metastasis^9,10^, leading to loss of cellular adhesion and tumor metastasis^8^. TNBC has a high rate of distant metastasis, and TNBC with mesenchymal features is highly malignant^11–13^. It has been suggested that suppressing the EMT may reduce the metastatic spread of TNBC. Recent studies on EMT focus on molecular levels, and it seems that the EMT does not simply rely on relative genetic alternation^14,15^. The EMT is a highly complex process triggered by diverse extracellular and intracellular signals via EMT-related transcription factors^16^.This may be because EMT is one of multiple steps during tumor metastasis, the purpose of which is to allow cancer cells to leave their primary location. Mesenchymal cancer cells undergo a mesenchymal-to-epithelial transition to form macro metastatic foci after extravasation in the distant organ.

MicroRNAs (miRNAs) are small noncoding RNAs that are involved in cell proliferation, survival, differentiation, and other essential cellular processes^17,18^. The abnormal expression of miRNAs has been detected in many diseases including cancer. Mounting evidence has reported the dysregulation of miRNAs in TNBC^19,20^. miRNAs are involved in the tumor initiation, progression, diagnostic, prognostic, and therapeutic potential of TNBC^21^. Moreover, they play important roles in regulating the EMT in breast cancer^22^. The miR-200 family, which inhibits EMT, tumor proliferation, migration, invasion, is downregulated in TNBC^22,23^. Similarly, miR-205, which is downregulated in TNBC, also can reduce proliferation and inhibit EMT^24^. Thus, regulating miRNA expression may inhibit the EMT, suggesting that using a single miRNA as an anti-cancer agent may provide high therapeutic efficacy without leading to “reactive resistance”. However, as an anti-cancer agent, the application of miRNA still has several challenges, such as unsatisfactory delivery efficacy.

In this study, we showed that miR-3178 was downregulated in TNBC. Ectopic overexpression of miR-3178 inhibited the EMT and metastasis in TNBC cells. Importantly, miR-3178 acted as a prognostic factor in TNBC but not in non-TNBC. With the aid of target predictors and a luciferase reporter assay, Notch1 was identified as the target gene of miR-3178. miR-3178 inhibited the expression of Notch1, and restoration of Notch1 expression reversed the inhibitory effects of miR-3178 on TNBC cell proliferation, migration, and the EMT. These results indicated that miR-3178 acted as a tumor suppressor in TNBC development by targeting Notch1. Thus, miR-3178 may serve as a powerful anti-cancer therapeutic agent with high efficacy.

## Results

### miR-3178 expression was significantly downregulated and acted as a prognostic factor in TNBC but not in non-TNBC

**Fig. 1:**
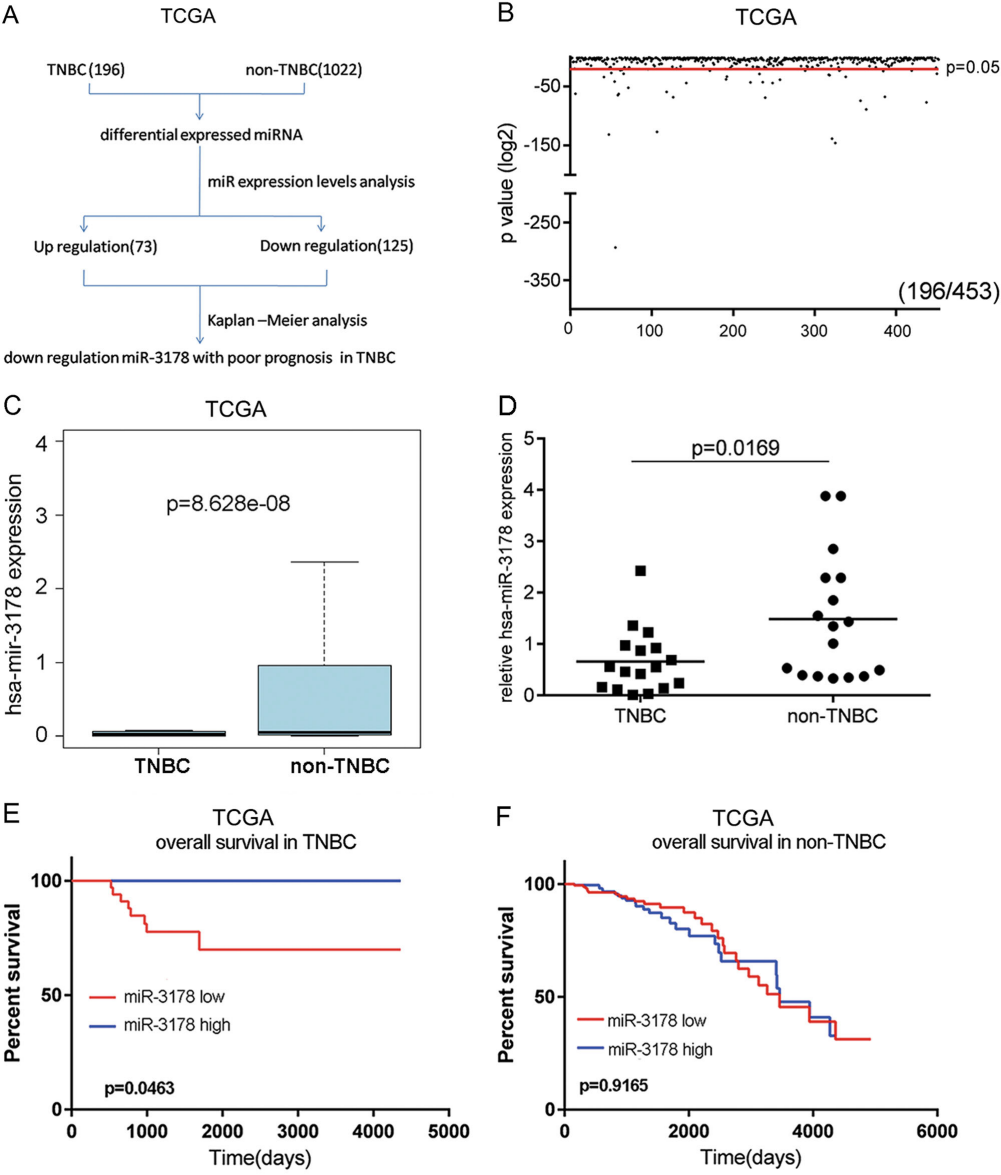
miR-3178 expression level was downregulated in TNBC. **a** The strategy for detecting different expression miRNAs between TNBC and non-TNBC in TCGA database. **b** Scatter plot of the different expression miRNAs. The points under the red line represent the differentially expressed miRNAs with statistical significance. **c** Expression levels of miR-3178 in TNBC and non-TNBC in TCGA. **d** Expression levels of miR-3178 in 17 TNBC and 17 non-TNBC tissues. **e**, **f** High levels of miR-3178 correlated with a better overall survival in TNBC. However, high miR-3178 expression was not associated with a better OS in non-TNBC

To identify the differential expression of miRNA between TNBC and non-TNBC, miRNA expression microarray analyses were performed using The Cancer Genome Atlas (TCGA) in 1283 breast cancer patients. The miRNA expression levels between 119 TNBC and 1164 non-TNBC patients were compared, and 196 miRNAs (72 upregulated and 124 downregulated) were found to be significantly different between the two groups (Fig. 1a, b). Among these miRNAs, miR-3178 was significantly lower in TNBC compared to the other subtypes (Fig. 1c). To verify the differences in miR-3178 expression, we performed qRT-PCR on 34 patient samples (17 with TNBC and 17 with non-TNBC), and found that miR-3178 was downregulated in TNBC (Fig. 1d). To further determine the prognostic significance of miR-3178, Kaplan–Meier analysis was used. Data on miR-3178 were obtained from 681 breast cancer patients from the TCGA database; 119 had TNBC and 562 had other subtypes (e.g., luminal A, luminal B, and HER-2). High miR-3178 expression contributed to better overall survival (OS) in stage I–IIIB TNBC (*n* = 66; 19 high expression and 47 low expression) (Fig. 1e). However, high miR-3178 expression was not associated with better OS in non-TNBC (Fig. 1f).

### miR-3178 suppressed the cell proliferation and migration of TNBC cells

**Fig. 2:**
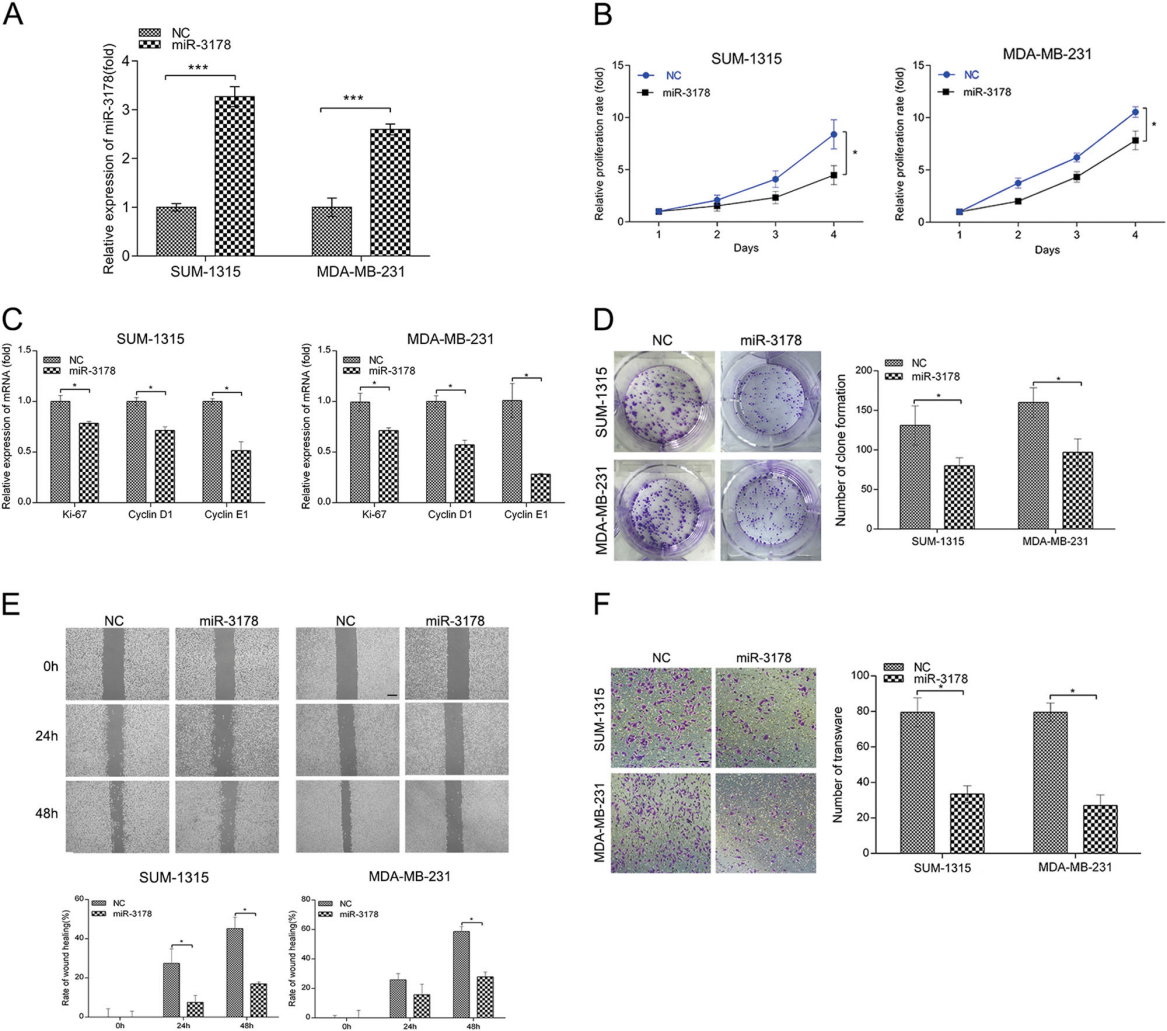
miR-3178 suppressed the cell proliferation and migration of TNBC cells. **a** Cells were transfected with miR-3178 mimic lentivirus, and the expression level of miR-3178 was detected after stable transfection. **b** Cell proliferation was evaluated by the CCK-8 assay at 24, 48, 72, and 96 h. **c** The effect of miR-3178 on mRNA expression levels of cell proliferation and cycle-related genes was detected by qRT-PCR. **d** Colony formation assay showed miR-3178 reduced colony numbers. The wound healing assay (**e**) and transwell invasion assay (**f**) were performed to detect the effects of miR-3178 on TNBC migration and invasion, scale bars: 300 μm (**e**) and 100 μm (**f**). ***P < 0.001, *P < 0.05

miR-3178 mimic lentivirus was transfected into two TNBC cell lines (MDA-MB-231 and SUM-1315), and the transfection efficiency was determined by qRT-PCR (Fig. 2a). The CCK-8 assay showed that the proliferation rate of TNBC cells transfected with miR-3178 mimics was significantly inhibited compared with the negative control group (NC) (Fig. 2b). The mRNA expression of the cell proliferation-associated marker Ki-67 and the cell cycle-related genes cyclinD1 and cyclin E1 was significantly reduced by miR-3178 mimics (Fig. 2c). Furthermore, the colony formation assay revealed that the colony number of TNBC cells transfected with miR-3178 mimics was significantly reduced compared with those transfected with NC (Fig. 2d). The wound healing assay and transwell invasion assay were performed to detect the effects of miR-3178 on TNBC migration and invasion (Fig. 2e, f). The results of the wound healing assay showed that miR-3178 mimics decreased TNBC cell migration; similar results were detected with the transwell invasion assay. The migratory and invasive abilities of TNBC cells transfected with miR-3178 mimics also decreased.

Further, miR-3178 inhibitor was transfected into SUM-1315 and MDA-MB-231 cells, and inhibited the expression of miR-3178 (Fig. S1A). The CCK-8 assay, wound healing assay and transwell invasion assay showed that suppression of miR-3178 can increase TNBC cell proliferation, migration and invasion, respectively (Fig S1B-D).

These results illustrated that miR-3178 inhibited the malignant characteristics of TNBC cells.

### miR-3178 inhibited the EMT in TNBC cells

**Fig. 3:**
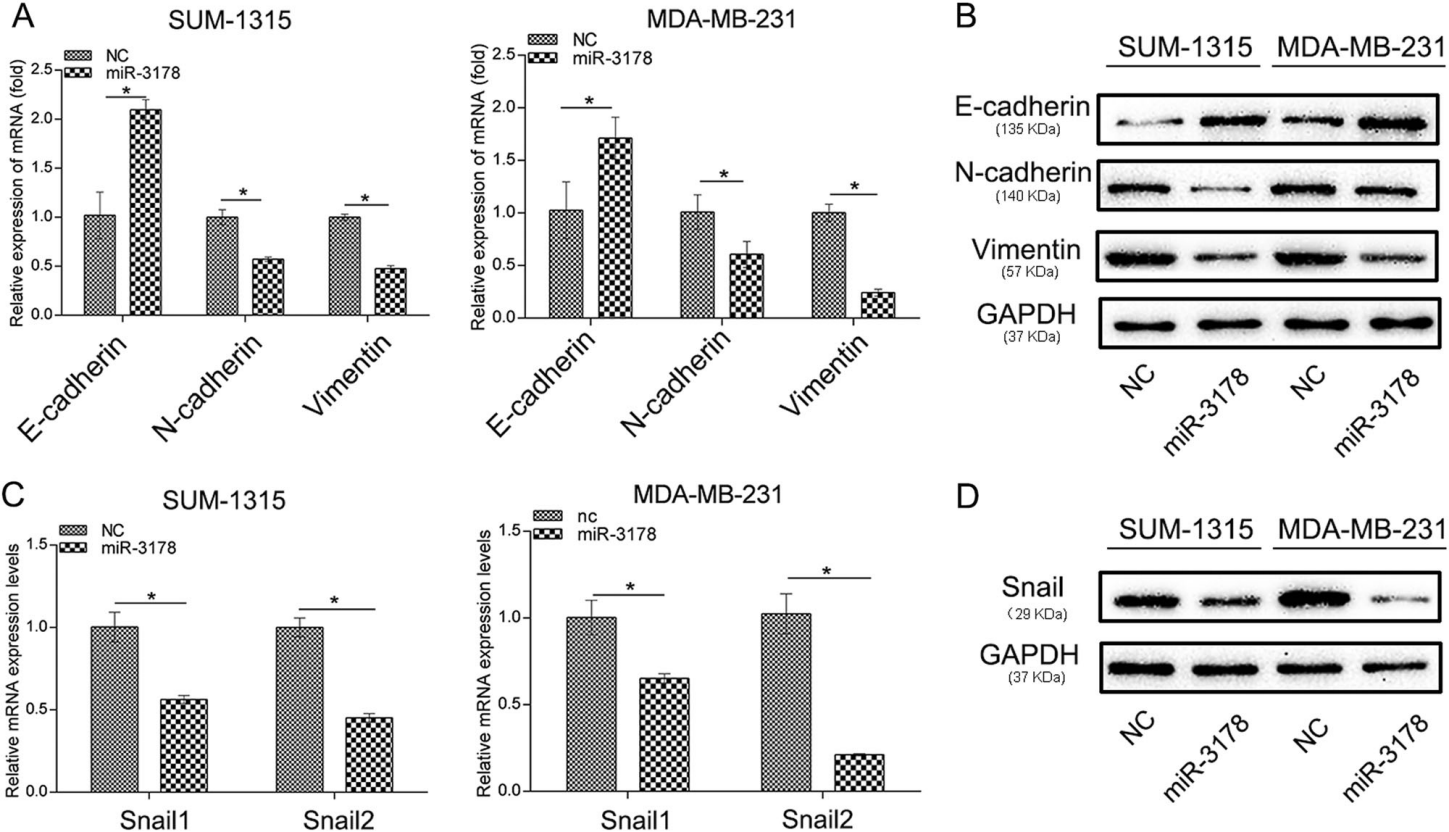
miR-3178 inhibited the EMT in TNBC cells. qRT-PCR (**a**) and western blot (**b**) analysis showed miR-3178 increased E-cadherin levels, and inhibited vimentin and N-cadherin levels in SUM-1315 and MDA-MB-231. **c** miR-3178 downregulated the mRNA expression levels of Snail and Slug, transcription factors that directly regulate the EMT. **d** Snail and Slug protein expression in SUM-1315 and MDA-MB-231 transfected with miR-3178 mimics. *P < 0.05

To determine the effects of miR-3178 on the EMT, the EMT markers were detected by qRT-PCR and western blotting. As shown in Fig. 3a, b, the epithelial cell marker of the EMT, E-cadherin, was significantly upregulated, but mesenchymal markers such as vimentin and N-cadherin were markedly downregulated in SUM-1315 and MDA-MB-231 cells transfected with miR-3178 mimics. Furthermore, miR-3178 also downregulated the mRNA and protein expression of Snail and Slug, transcription factors that directly regulate the EMT (Fig. 3c, d). This further supports the hypothesis that miR-3178 reduces tumor malignancy by inhibiting the EMT in TNBC.

### Notch1 was a direct downstream target of miR-3178

**Fig. 4:**
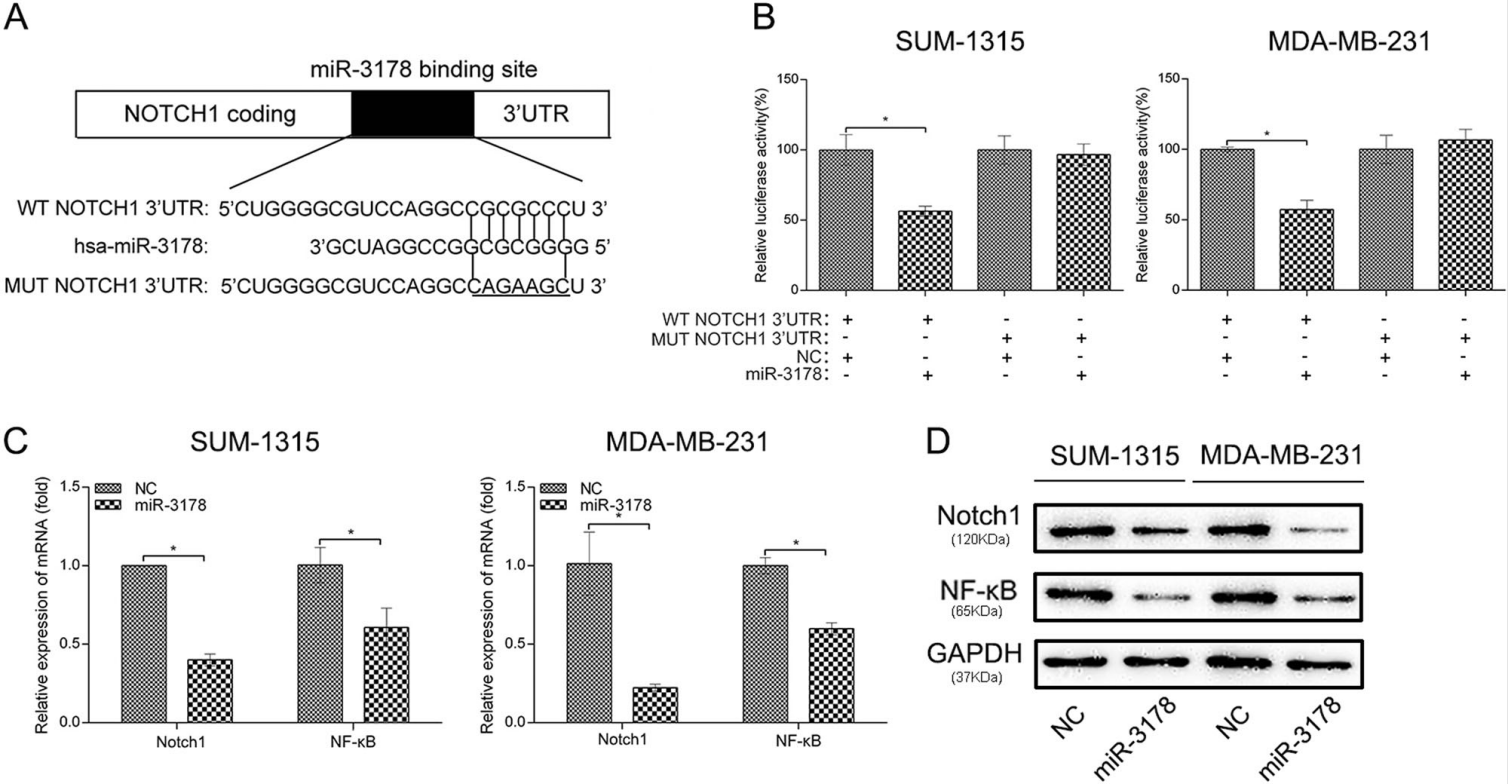
Notch1 was a direct downstream target of miR-3178. **a** The luciferase reporter constructs that had either a WT-notch1-3′-UTR or mut-notch1-3′-UTR sequence of the miR-3178-binding site. **b** Luciferase reporter activity revealed miR-3178 suppressed Notch1 3′UTR luciferase activity of wide-type constructs in SUM-1315 and MDA-MB-231 cells. **c** qRT-PCR analysis showed that miR-3178 mimics decrease Notch1 and NF-κB (a downstream factor of Notch1) mRNA level. **d** Notch1 and NF-κB protein expression in SUM-1315 and MDA-MB-231 transfected with miR-3178 mimics. * *P* < 0.05

The miR data bases, TargetScan and miRNATar Base were used to predict the target genes of miR-3178 that may regulate tumor growth and metastasis, and Notch1 was recognized as a potential target. Then, the luciferase assay was performed to determine whether there was a direct correlation between miR-3178 and Notch1. We constructed luciferase reporter constructs that had either a WT-notch1-3′-UTR or mut-notch1-3′-UTR sequence of the miR-3178-binding site (Fig. 4a). The relative luciferase activity was decreased after co-transfection with miR-3178 and WT-notch1-3′-UTR in MDA-MB-231 and SUM-1315 cells. The decreased luciferase activity was not checked after transfected miR-3178 with mut-notch1-3′-UTR (Fig. 4b). To further support the specific regulation of Notch1 by miR-3178, we measured Notch1 expression in miR-3178 mimic-transfected cells by qRT-PCR and western blot analysis. The overexpression of miR-3178 decreased Notch1 mRNA and protein expression, and NF-κB (a downstream factor of Notch1) was also inhibited by miR-3178 (Fig. 4c, d).

### Restored expression of Notch1 attenuated inhibition of cancer cell proliferation by miR-3178 in TNBC cells

si-Notch1 was transfected into SUM-1315 and MDA-MB-231 cells to confirm the effects of miR-3178 on proliferation, migration and invasion in TNBC were mediated by downregulation of Notch1. Then we utilized the qRT-PCR and western blot to analysis the downexpression of Notch1 (Fig. S2A, B). The CCK-8 assay, wound healing assay and transwell invasion assay were also performed, and we found that inhibition of Notch1 could suppress the proliferation, migration, and invasion in SUM-1315 and MDA-MB-231 cells (Fig. S2C-E).

**Fig. 5:**
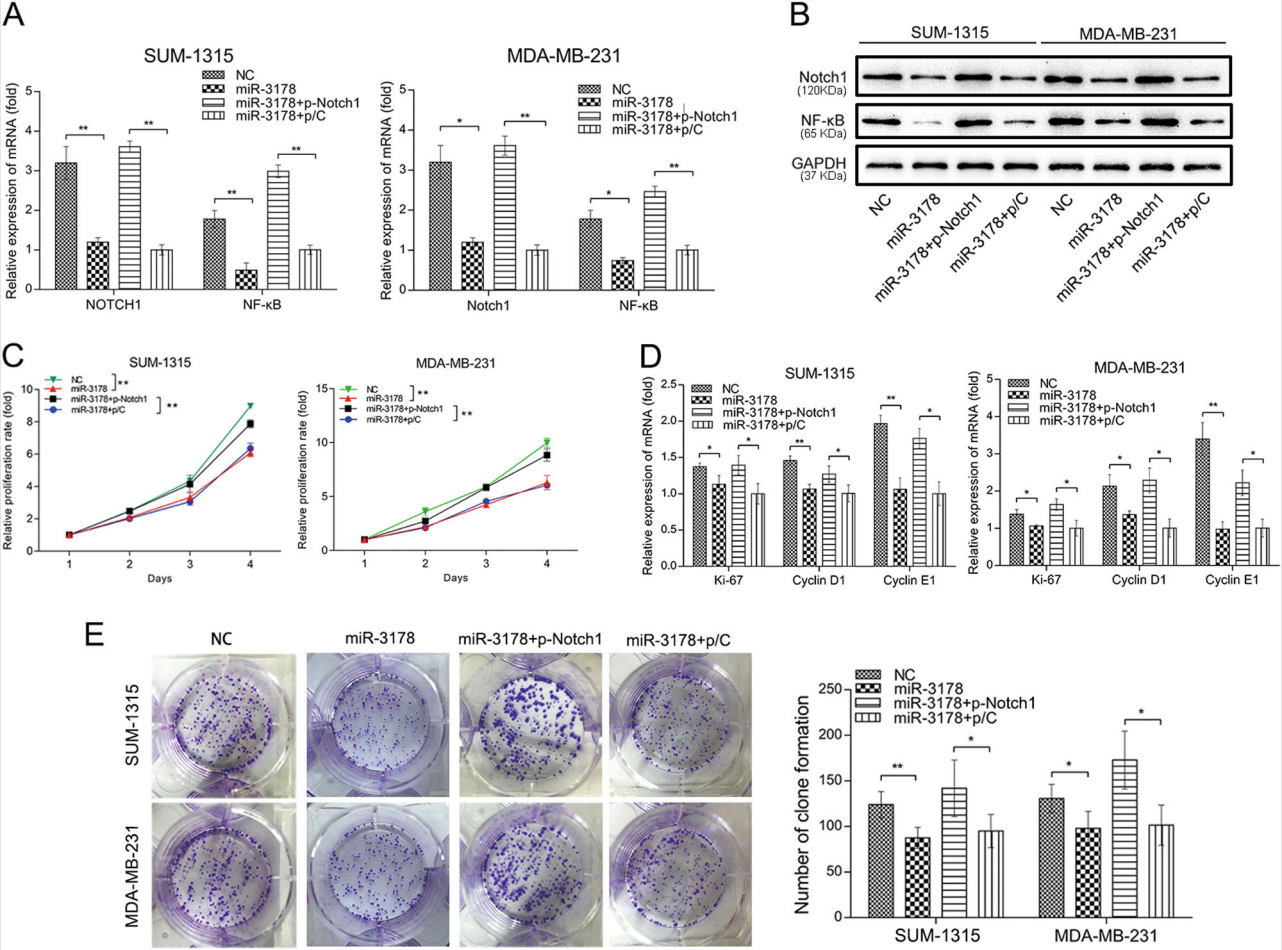
Expression of Notch1 attenuated inhibition of cancer cell proliferation by miR-3178 in TNBC cells. Cells were transfected with Notch1 overexpression plasmid (p-Notch1), and the mRNA and protein expression of Notch1 and NF-κB protein level was detected by qRT-PCT (**a**) and western blot (**b**). **c** CCK8 assay showed the inhibition of cell proliferation by miR-3178 was reversed by p-Notch1. **d** p-Notch1restoredthe mRNA levels of Ki-67, cyclinD1, and cyclin E1, which were inhibited by miR-3178 mimics. **e** p-Notch1 reversed the inhibitory effects of miR-3178 on colony formation. **P < 0.01, *P < 0.05

To further investigate whether miR-3178 exerts its anti-tumor effects by targeting Notch1, Notch1 expression was upregulated by Notch1 plasmid (p-Notch1). As shown in Fig. 5a, b, Notch1 and NF-κB were downregulated by miR3178 mimics in SUM-1315 and MDA-MB-231 cells, which was reversed with transfection of p-Notch1. Fig. 5c shows that the inhibition of cell proliferation by miR-3178 was also reversed by p-Notch1. Furthermore, p-Notch1 restored the mRNA levels of Ki-67, cyclinD1, and cyclin E1, which were inhibited by miR-3178 mimics (Fig. 5d). In addition, p-Notch1 reversed the inhibitory effects of miR-3178 on colony formation (Fig. 5e).

### miR-3178 regulated Notch1-mediated migration and the EMT through Snail1 in TNBC cells

**Fig. 6:**
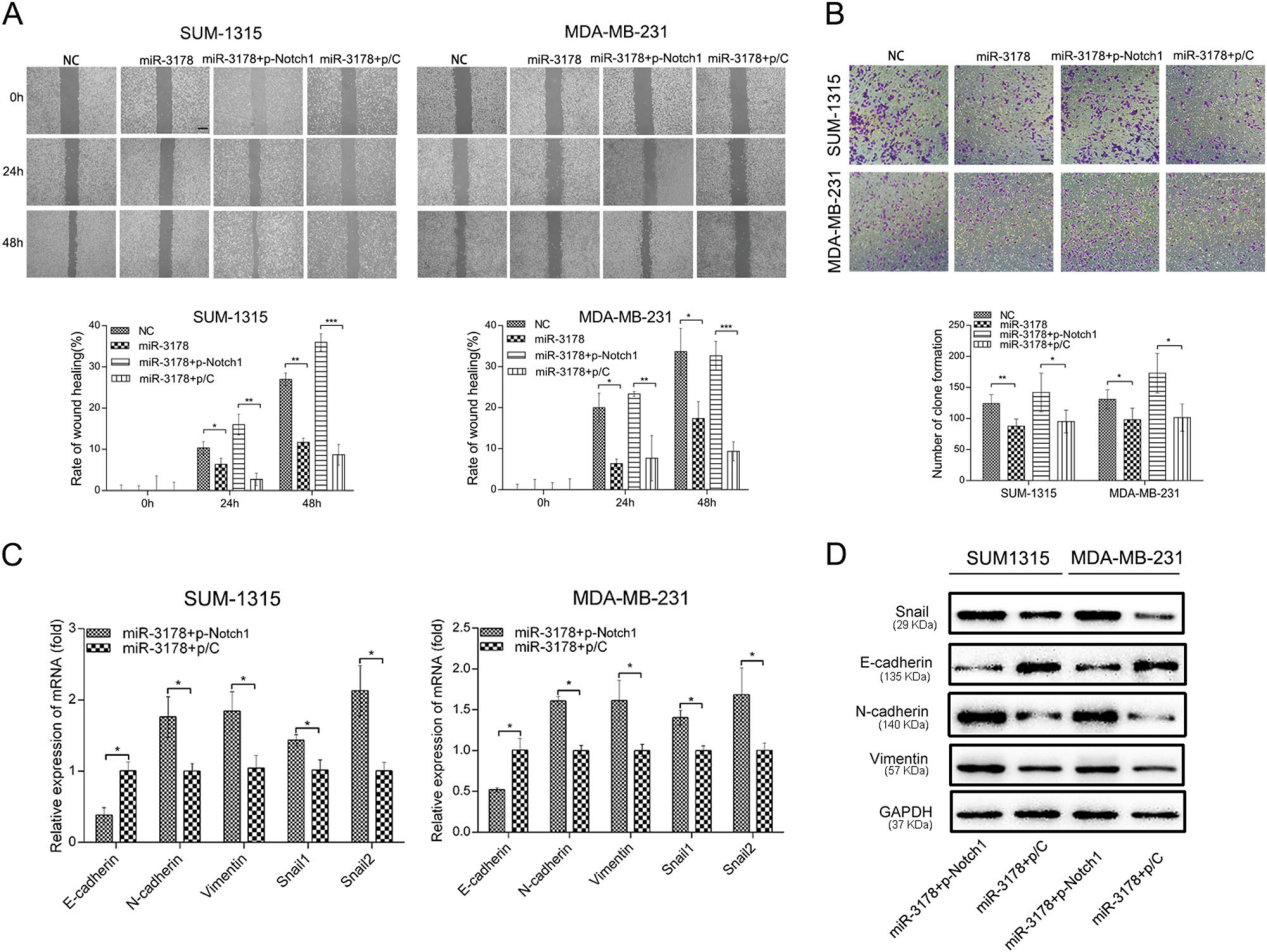
miR-3178 regulated Notch1-mediated migration and the EMT through Snail1 in TNBC cells. The wound healing (**a**) and transwell assay (**b**) showed the inhibition of miR-3178 on cell migration and invasion was attenuated after restoring the expression of Notch1 by p-Notch1, scale bars: 300 μm (**a**) and 100 μm (**b**). **c** p-Notch1 reversed the effect of miR-3178 on the mRNA expression of the EMT markers and Snail. **d** p-Notch1 restored the protein expression of the EMT markers and Snail. *** *P* <0.001, ** P <0.01, * *P* < 0.05

Studies have shown that Notch1 is an important regulator of the EMT and is associated with tumor migration and invasion in breast cancer. As shown in Fig. 6a, b, restoring the expression of Notch1 by p-Notch1 attenuated the inhibition of miR-3178 on cell migration and invasion. To further confirm that miR-3178 regulated the EMT by targeting Notch1, the Notch1 downstream factor Snail1 was detected by qRT-PCR and western blot analysis. After increasing the activity of Notch1 by p-Notch1, the expression of Snail1 was upregulated compared to expression in miR-3178 mimic-transfected cells. Expression of the EMT markers affected by miR-3178 mimics was also reversed by p-Notch1 (Fig. 6c, d).

### miR-3178 inhibited tumor growth in a nude mouse model of TNBC

**Fig. 7:**
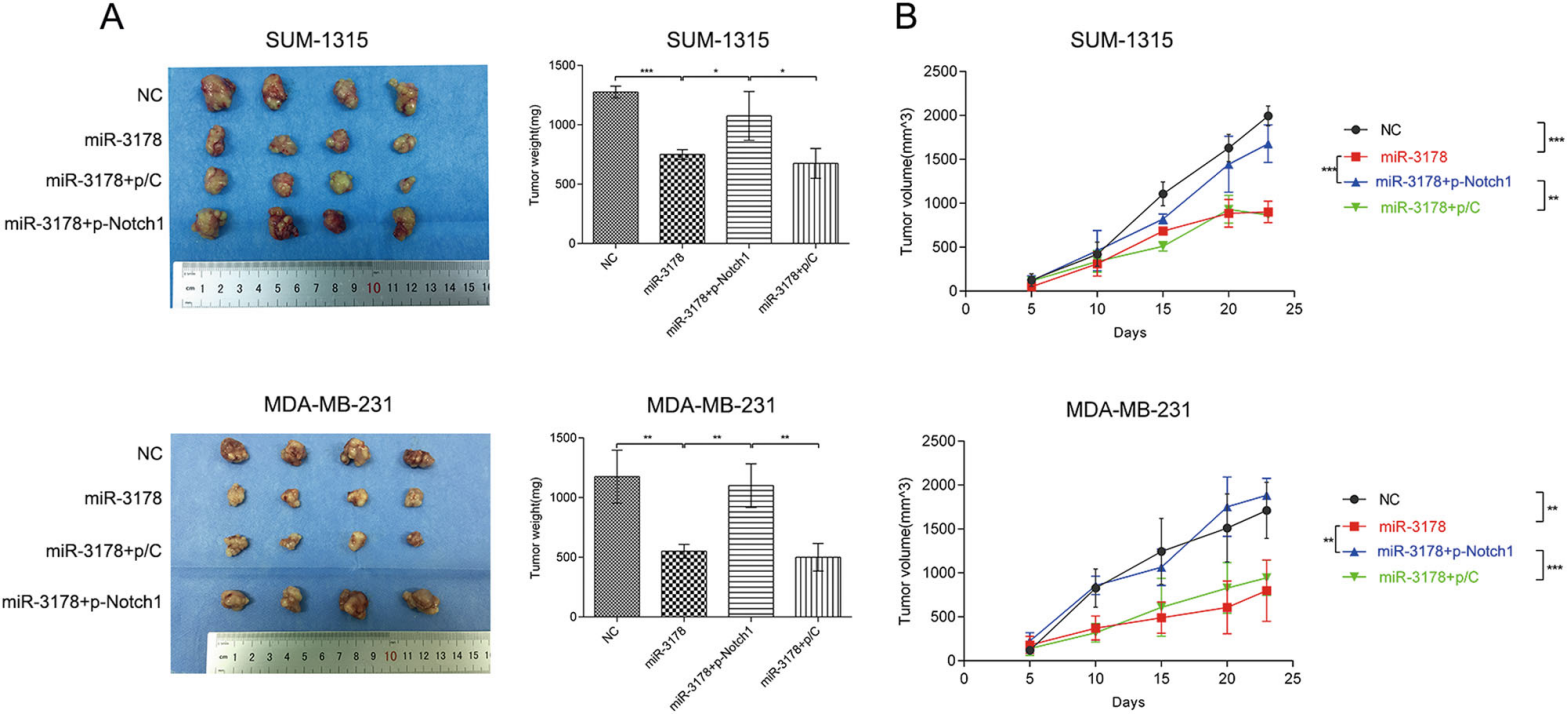
miR-3178 inhibited tumor growth in a nude mouse model of TNBC. **a** MDA-MB-231 and SUM-1315 cells transfected with miR-3178, miR-3178/p-Notch1, miR-3178/p-NC, and m-NC were subcutaneously implanted into nude mice. The tumor weight of miR-3178 and miR-3178/p-NC was lower than that with m-NC and miR-3178/p-Notch1. **b** miR-3178 significantly decreased tumor volume compared with m-NC. miR-3178/p-Notch1 partly neutralized the inhibitory effects of miR-3178 on tumor volume. ***P *<* 0.001, **P *<* 0.01, *P < 0.05

To assess the effects of miR-3178 on tumor growth in vivo, miR-3178, miR-3178/p-Notch1, miR-3178/p-NC, and m-NC MDA-MB-231 and SUM-1315 cells were subcutaneously implanted into nude mice. Tumor volume and weight were recorded. The tumor weight of miR-3178 and miR-3178/p-NC was significantly lower than that with m-NC and miR-3178/p-Notch1 (Fig. 7a). Same as the cell proliferation observed in vitro, miR-3178 significantly decreased tumor volume compared with m-NC. miR-3178/p-Notch1 partly neutralized the inhibitory effects of miR-3178 on tumor volume (Fig. 7b).

## Discussion

Due to a lack of treatment targets, chemotherapy has remained the main therapeutic strategy for TNBC. TNBC has high pathologic complete response rates after neoadjuvant chemotherapy^25,26^, but it also has more chemoresistance and a worse prognosis than other breast cancer subtypes^2,3,27^. Thus, it is necessary to find novel targets for TNBC. The current possible targets for TNBC are mainly aberrant signal transduction pathways or overexpressed proteins in TNBC^28–33^, such as mTOR inhibitors, Src tyrosine kinase inhibitors, PARP inhibitors, anti-EGFR, and anti-VEGFR therapies. The abnormal expression of miRNAs correlates with tumor initiation and progression in TNBC^21^. The reason of miRNAs reduction may be relative promoter hypermethylation in TNBC^34^. Besides, Long noncoding RNAs (LncRNAs) could inhibit miRNAs directly in TNBC^35^. miRNAs, small noncoding RNAs, bind to the 3′UTR on target mRNAs, leading to mRNA degradation and inhibition of protein translation. A single miRNA can regulate multiple target genes, which makes miRNAs large and powerful regulators of tumor genesis and progression^36^. In our study, miR-3178 was downregulated in TNBC compared with other subtypes of breast cancer. The ectopic overexpression of miR-3178 significantly reduced the proliferation and migration of MDA-MB-231 and SUM-1315 TNBC cells, and similar results were observed in the animal models. These results demonstrate the anti-tumor characteristics of miR-3178 in TNBC.

To illustrate the inhibition of miR-3178 in TNBC, the underlying mechanisms were analyzed. The miRNAs exhibited anti-tumor effects mainly through suppressing proliferation and migration and promoting apoptosis in TNBC^37–39^. TNBC has more distant metastasis than other subtypes of breast cancer, and several studies have confirmed that metastasis is more likely to occur in TNBC with mesenchymal characteristics^13,40^. Thus, reversing the EMT may be a possible strategy for treating TNBC. The EMT is a fundamental mechanism for embryo development, wound healing, and organ fibrosis, and is also the initial event of tumor metastasis. The EMT is a highly complex process that is regulated by the extracellular matrix, autocrine, or paracrine cell factors^16^, as well as by miRNAs^41^, which are associated with the EMT and metastasis of breast cancer^19,42^. Our data indicated that mesenchymal characteristics were reduced after the upregulation of miR-3178 in TNBC cells. Analysis of the database showed that Notch1 was a potential target gene for miR-3178. Furthermore, miR-3178 directly combined with Notch1 and inhibited its expression. This further supports the hypothesis that miR-3178 inhibits the EMT by targeting Notch1 in TNBC.

The Notch family is important for the intercellular communication system, and is involved in cell differentiation, proliferation, death, and cancer initiation^43,44^. In solid tumors, Notch signaling regulates tumor growth, cell survival, angiogenesis, cell differentiation, and development. Besides, Notch signaling plays an important role in the development of cancer stem cells (CSCs)^45^, and maintains CSCs stemness, self-renewal and proliferation^46,47^. The abnormal expression of Notch has also been detected in TNBC^48,49^, and a phase I trial of RO4929097 (notch/secretase inhibitor) is currently investigating Notch1 in combination with neoadjuvant chemotherapy (paclitaxel and carboplatin) for inoperable TNBC. Notch1, one of four Notch receptors, is overexpressed in TNBC^48^, indicating that it could be a therapeutic target in TNBC. In our study, miR-3178 significantly reduced the expression of Notch1, and the restored expression of Notch1 attenuated the inhibition of miR-3178 on tumor proliferation and metastasis.

In conclusion, the results of this study showed that miR-3178 was downregulated and acted as a tumor suppressor by reducing cell growth and metastasis in TNBC. Importantly, miR-3178 acted as a prognostic factor in TNBC but not in non-TNBC. These anti-tumor effects of miR-3178 were dependent on its direct regulation of Notch1. These data may provide a promising approach for the treatment of TNBC.

## Methods

### Cell culture and animals

Two human TNBC cell lines were used in this study: MDA-MB-231 was obtained from America Type Culture Center (ATCC, Manassas, VA, USA), and SUM-1315 was kindly provided by Stephen Ethier (University of Michigan, Ann Arbor, MI, USA). The cells were cultured in Dulbecco’s modified Eagle medium (DMEM, GIBCO, Suzhou, China) containing 10% fetal bovine serum and 1% penicillin-streptomycin, and maintained at 37 °C with 5% CO_2_.

### Patient tissue samples

The breast cancer tissues and adjacent normal tissues were obtained from the First Affiliated Hospital with Nanjing Medical University. The breast cancer patients did not receive any chemotherapy or endocrine therapy before tumor resection. All tissues were frozen in liquid nitrogen immediately and stored at −80 °C after surgical resection. Informed consent was obtained from each patient, and this study was approved by the Ethics Committees of the First Hospital Affiliated Hospital with Nanjing Medical University.

### Transfection of lentivial vectors in vitro

Cells in which miR-3178 was overexpressed were generated by transfecting miR-3178 mimics. A total of 2 × 10^5^ breast cancer cells/well in 6-well plates were grown overnight and transduced by adding 8 µg/mL polybrene and 10 µL lenti-miR-3178 and lenti-miR-Scramble lentiviral particles (LV5-EF1a-GFP/Puro; Shanghai GenePharma Co., Shanghai, China). After 24 h, stably transfected cells were selected using 0.3 µg/mL puromycin for 4 weeks.

### Animal models

Animal experiments were approved by the Institutional Animal Care and Use Committee, Nanjing Medical University (Nanjing, China). Female Balb/c nude mice (4–6 weeks old) were purchased from the Model Animal Research Center of Nanjing University and maintained under specific pathogen-free conditions in the Animal Core Facility of Nanjing Medical University. A total of 2 × 10^6^ cells were injected on the left side of the second pair of mammary fat pads of nude mice. Tumor volume was detected every 5 days and calculated as follows: total tumor volume (mm^3^) = length × width^2^/2. Weight loss was monitored every 5 days. Mice were sacrificed when their weight decreased by 30%. At 25 days after injection, the tumor specimens were removed, weighed, and fixed in 10% buffered formalin for further analysis.

### Cell proliferation, colony formation, migration, and invasion assay in vitro

A total of 3 × 10^3^/well cells were seeded in 96-well plates, and cell proliferation was detected using Cell Counting Kit-8 (CCK-8; Dojindo Laboratories, Kumamoto, Japan) according to the manufacturer’s instructions. To evaluate colony formation, the colony formation assay was performed. Briefly, 500 cells per well were seeded into 6-well plates and incubated for about 2 weeks. Then, the plates were washed three times with phosphate-buffered saline and stained with crystal violet (Beyotime, Shanghai, China). The number of cell colonies formed per well was counted. For the cell migration assay, the wound healing test was performed. To this end, 3 lines of 1 cm intervals were drawn behind 6-well plates. Then, 6 × 10^5^ transfected cells were inoculated into the 6-well plates and incubated for 24 h. For the scratch test, when the cell density reached 80–90%, scratching the cells with the lines perpendicular to the previously painted line. Three fields were randomly selected from each well, and cell movements in the scratch were observed and photographed at 0, 24, and 48 h. The width of the scratch was detected with Image Pro 6.0 software. These experiments were repeated at least three times. The transwell assays (Minicell, Millipore, USA) was used to quantify cell invasion. The transwell membranes were pre-coated with 50 μL matrigel (Corning Inc., Corning, NY, USA), and 200 μL cell suspension (5 × 10^3^ cells) in serum-free DMEM was added to the upper chamber with 8.0 μm pore size membrane inserts in the 24-well plates. Cells that migrated to the outside of the membrane were stained with crystal violet (Beyotime) after 24 h. The cell number was counted at a ×200 magnification in five randomly selected regions per well.

### Luciferase reporter assay

Possible miR-3178 binding sites were obtained from a miRNA database (targetscan.org). Wild-type Notch1 (WT-notch1-3′-UTR) and mutant Notch1 (mut-notch1-3′-UTR) were purchased from Shanghai Genechem Co., Ltd. (Shanghai, China). For the luciferase reporter assay, cells were co-transfected with WT-notch1-3′-UTR or mut-notch1-3′-UTR plasmids and miR-3178. Firefly luciferase activities were measured using the Dual Luciferase Assay System (Promega, Madison, WI, USA) 48 h after transfection, and the results were normalized with Renilla luciferase.

### Quantitative real-time PCR

Total mRNA was extracted using TRIzol reagent (Invitrogen, Carlsbad, CA, USA). Quantitative real-time PCR (qRT-PCR) for miRNA detection was performed with the indicated TaqMan MicroRNA Assay (Thermo Fisher Scientific, Waltham, MA, USA) according to manufacturer’s instructions, and normalized to U6 RNA levels. Reverse transcription of total RNA was performed using an RT-PCR Kit (TaKaRa, Otsu, Japan), according to previously described procedures^50^. The primer sequences used for detecting target genes are shown in Table 1.

**Table 1:** The sequence of primer

Primer	Sequence
GAPDH	
Fwd	5′-GAAGGTGAAGGTCGGAGTC-3′
Rev	5′- GAAGATGGTGATGGGATTTC-3′
Ki-67	
Fwd	5′-ACGCCTGGTTACTATCAAAAGG-3′
Rev	5′- CAGACCCATTTACTTGTGTTGGA-3′
Cyclin D1	
Fwd	5′-GCTGCGAAGTGGAAACCATC-3′
Rev	5′-CCTCCTTCTGCACACATTTGAA-3′
Cyclin E1	
Fwd	5′-ACTCAACGTGCAAGCCTCG-3′
Rev	5′-GCTCAAGAAAGTGCTGATCCC-3′
CDH1	
Fwd	5′- CAGCACGTACACAGCCCTAA-3′
Rev	5′- TGAGGCTTTGGATTCCTCTC-3′
CDH2	
Fwd	5′- CAGCACGTACACAGCCCTAA-3′
Rev	5′- TGAGGCTTTGGATTCCTCTC-3′
vimentin	
Fwd	5′- CAGATGCGTGAAATGGAAGA-3′
Rev	5′- CTCAATGTCAAGGGCCATCT-3′
Snail1	
Fwd	5′-CCTCCACGAGGTGTGACTAACT-3′
Rev	5′-CCGACAAGTGACAGCCATTA -3′
Snail2	
Fwd	5′-CGCAATCAATGTTTACTCGAAC-3′
Rev	5′- TCTCAATCTAGCCATCAGCAAA-3′
Notch1	
Fwd	5′-ATCGACATGGCCGAATGGAA-3′
Rev	5′-ATGATGTCCACGCCCTTCTG-3′
NF-κB	
Fwd	5′-GGTGCGGCTCATGTTTACAG-3′
Rev	5′-GATGGCGTCTGATACCACGG-3′
miR-3178	
Fwd	5′-GGGGCGCGGCCGGATCG-3
Rev	5′-GCTGTCAACGATACGCTACGTAACG-3′
U6	
Fwd	5′-CTCGCTTCGGCAGCACA-3′
Rev	5′-AACGCTTCACGAATTTGCGT-3′

### Western blot analysis

Western blot analysis was performed as previously described^51^. The antibodies used were as follows: anti-E-cadherin (Cell Signaling Technology [CST], Danvers, MA, USA), anti-N-cadherin (CST), anti-Snail (CST), anti-NF-κB (CST), anti-vimentin (Abcam, Cambridge, MA, USA), anti-Notch1 (Abcam) and anti-GAPDH (Beyotime).

### Statistics

Numerical data are presented as mean ± standard deviation. All of the data were statistically analyzed by the Student’s *t*-test or one-way analysis of variance. IBM SPSS Statistics version 22 (Chicago, IL, USA) was used, and *p* < 0.05 was considered statistically significant.

## Electronic supplementary material

Fig S1. miR-3178 suppressed the cell proliferation, migration and invasion of TNBC cells

Fig S2. Notch1 promotes the cell proliferation, migration and invasion of TNBC cells
