# Supplementary material for: miR-3178 inhibits cell proliferation and metastasis by targeting Notch1 in triple-negative breast cancer
Source: Cell Death Dis. 2018 Oct 17;9(11):1059. doi: 10.1038/s41419-018-1091-y (PMC6192997; doi:10.1038/s41419-018-1091-y)
Supplement: Supplementary file 1 — Fig S1. miR-3178 suppressed the cell proliferation, migration and invasion of TNBC cells [file 41419_2018_1091_MOESM1_ESM.docx]

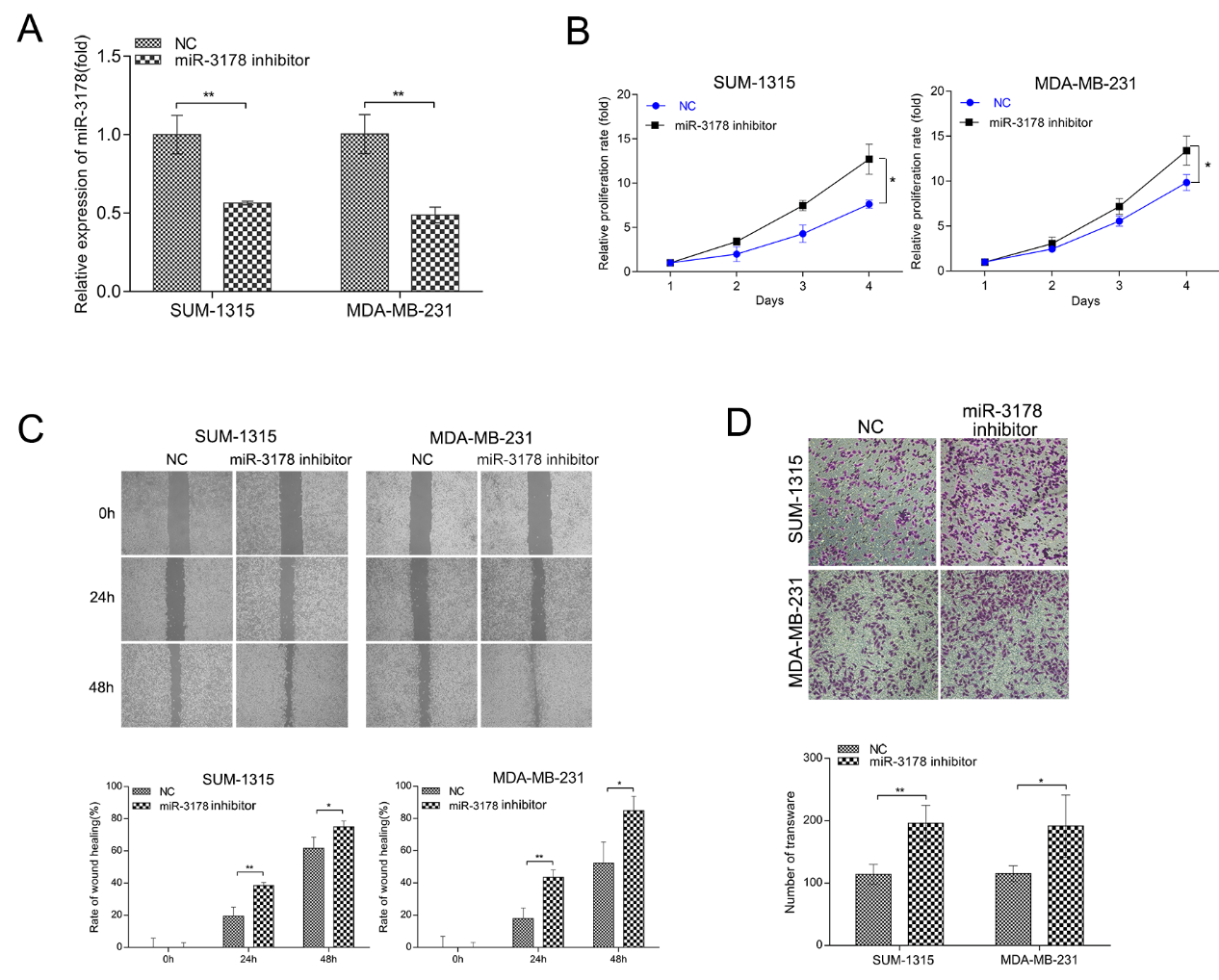


**Fig S1 miR-3178 suppressed the cell proliferation, migration and invasion of TNBC cells.** (A) Cells were transfected with miR-3178 inhibitor, and the expression level of miR-3178 was detected by qRT-PCR analysis. (B) Cell proliferation was evaluated by the CCK-8 assay at 24, 48, 72 and 96h. Wound healing assay (C) and transwell invasion assay (D) and were performed to detect the effects of miR-3178 on TNBC migration and invasion. **P < 0.01, *P<0.05
