# Supplementary material for: miR-3178 inhibits cell proliferation and metastasis by targeting Notch1 in triple-negative breast cancer
Source: Cell Death Dis. 2018 Oct 17;9(11):1059. doi: 10.1038/s41419-018-1091-y (PMC6192997; doi:10.1038/s41419-018-1091-y)
Supplement: Supplementary file 2 — Fig S2. Notch1 promotes the cell proliferation, migration and invasion of TNBC cells [file 41419_2018_1091_MOESM2_ESM.docx]

**
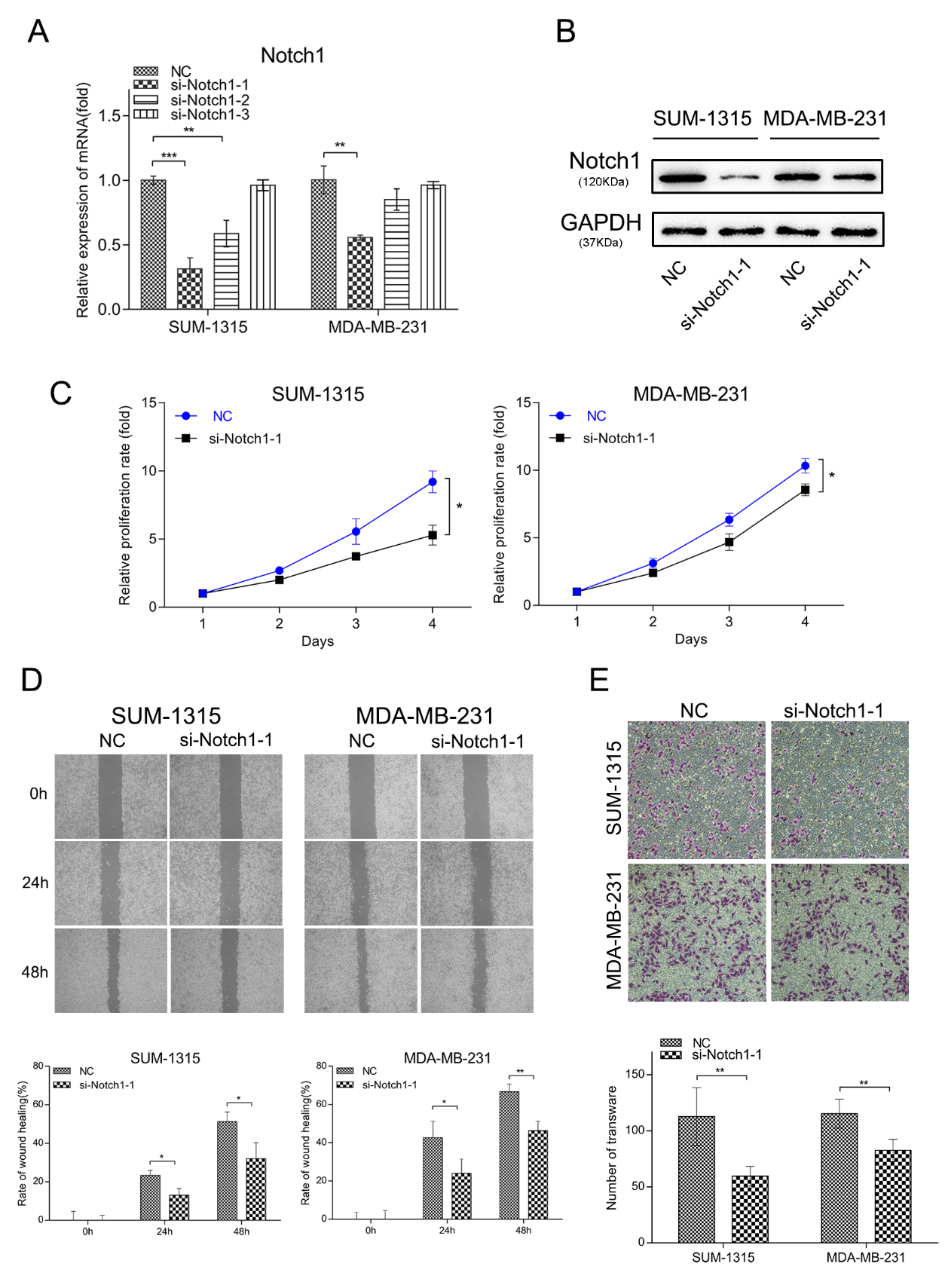
**

**Fig S2 Notch1 promotes the cell proliferation, migration and invasion of TNBC cells.** Cells were transfected with si-Notch1, and the expression level of Notch1 was detected by qRT-PCR analysis (A) and western blot (B). (C) Cell proliferation was evaluated by the CCK-8 assay at 24, 48, 72 and 96h. Wound healing assay (D) and transwell invasion assay (E) and were performed to detect the effects of Notch1 on TNBC migration and invasion. ***P<0.001, **P < 0.01, *P<0.05
